# Early childhood feeding practices and dental caries in preschool children: a multi-centre birth cohort study

**DOI:** 10.1186/1471-2458-11-28

**Published:** 2011-01-12

**Authors:** Amit Arora, Jane A Scott, Sameer Bhole, Loc Do, Eli Schwarz, Anthony S Blinkhorn

**Affiliations:** 1 Department of Population Oral Health, Faculty of Dentistry, The University of Sydney, Sydney, Australia; 2 Nutrition and Dietetics, School of Medicine, Flinders University, Adelaide, Australia; 3 Sydney Dental Hospital and Oral Health Services, Sydney South West Area Health Service, New South Wales, Sydney, Australia; 4 Australian Research Centre for Population Oral Health, The University of Adelaide, Adelaide, Australia

## Abstract

**Background:**

Dental caries (decay) is an international public health challenge, especially amongst young children. Early Childhood Caries is a rapidly progressing disease leading to severe pain, anxiety, sepsis and sleep loss, and is a major health problem particularly for disadvantaged populations. There is currently a lack of research exploring the interactions between risk and protective factors in the development of early childhood caries, in particular the effects of infant feeding practises.

**Methods/Design:**

This is an observational cohort study and involves the recruitment of a birth cohort from disadvantaged communities in South Western Sydney. Mothers will be invited to join the study soon after the birth of their child at the time of the first home visit by Child and Family Health Nurses. Data on feeding practices and dental health behaviours will be gathered utilizing a telephone interview at 4, 8 and 12 months, and thereafter at 6 monthly intervals until the child is aged 5 years. Information collected will include a) initiation and duration of breastfeeding, b) introduction of solid food, c) intake of cariogenic and non-cariogenic foods, d) fluoride exposure, and e) oral hygiene practices. Children will have a dental and anthropometric examination at 2 and 5 years of age and the main outcome measures will be oral health quality of life, caries prevalence and caries incidence.

**Discussion:**

This study will provide evidence of the association of early childhood feeding practices and the oral health of preschool children. In addition, information will be collected on breastfeeding practices and the oral health concerns of mothers living in disadvantaged areas in South Western Sydney.

## Background

Dental caries (decay) is one of the most prevalent chronic childhood diseases worldwide and is a major problem both from a population health perspective and for individual families who have to deal with a young child suffering from toothache [1-3]. In 1996, 39 percent of Australian 6 year-old children had dental caries [4,5], and since that time caries experience in Australian children in all States and Territories has increased [5,6]. The 2002 Child Dental Health Survey of Australia reported that 45 percent of 5-year-olds had one or more decayed or missing teeth and 10 percent of those children examined were found to have more than seven decayed teeth [7]. Local data from the Centre for Oral Health Strategy (NSW Health) indicates that despite water fluoridation, dental caries is a major public health problem particularly in disadvantaged areas. For example, 40 percent of the 5-6-year-olds [8] surveyed in the Sydney South West Area Health Service (SSWAHS) had up to 5 missing or decayed teeth- a huge burden of disease, when one considers there are only 20 baby teeth in the whole mouth.

The problem is not unique to Australia. In the United Kingdom successive national child dental health surveys have shown little change in caries prevalence in five-year-old children over the last 20 years [9]. Data from the United States of America tells a similar story; from 1988-1994 and 1999-2002 there was no change in the prevalence of dental caries among children aged 2-11 years [10]. These trends in oral health are important in dispelling the myth that oral health problems are largely solved and that future investments in dental care and health promotion are not warranted. For example, Early Childhood Caries (ECC) in children aged less than 71 months is a rapidly progressing disease leading to childhood distress [11], repeat prescriptions of antibiotics [12], severe pain, sepsis, and sleep loss [13].

Many dental services are overwhelmed with preschool children with severe dental problems who for many, as they are young and uncooperative, treatment under General Anaesthesia (GA) is the only viable option to ensure painful teeth are removed [14]. Hospitalisation rates for the removal or restoration of decayed teeth among New South Wales (NSW) children under 5 years has increased by 90 percent between 1989 and 2007 [15]. As a consequence of the need for GA the waiting lists are long (over 1 year) and the cost per case in Australia is AU$2011 [16]. In the year 2006-2007, 1720 children aged between 0-4 years received dental treatment under GA at a cost of approximately 3.5 million Australian dollars, excluding the preliminary out-patient costs [17]. These data highlight the fact that dental caries is the most costly diet related chronic disease [18] ahead of coronary artery disease, overweight and/or obesity, hypertension and diabetes. This places a huge financial burden on the hospital services and the long waiting lists cause considerable distress to both parents and young children.

Australia's National Oral Health Plan [19] highlighted this anomaly and called for more evidence based research into the prevention of dental disease; especially in younger children, as those with caries in the early years are more likely to have compromised adult teeth and will consume even more treatment resources later in life. Estimates from the United States, which has a similar privately funded dental system to Australia, indicate that the overall cost of restoring a permanent tooth is approximately US$ 1811 over a person's lifetime [20].

Fisher-Owens et al [21] reviewed the research evidence on early childhood caries in some detail, and they suggested that disease initiation depended on an interaction between family and community factors. In addition, it has been reported that early colonization of Streptococcus Mutans, plaque accumulation, and behavioural habits are strongly associated with ECC [22]. However, the effect of some feeding practices such as breast-feeding on the development of dental caries remains unclear and further research is needed. Furthermore, the relationship between overweight and/or obesity and ECC is ambiguous [23].

While the scientific basis of the messages to promote breastfeeding for general health are well accepted, there appears to be a clinical consensus amongst dental practitioners that prolonged and nocturnal breastfeeding is associated with an increased risk of ECC. However, the evidence for such an association of is limited and inconsistent, and is based primarily on cross-sectional studies relying on retrospective recall of infant feeding practices [24-28]. Furthermore, these studies and subsequent longitudinal studies have failed to adequately measure and control for confounding variables in their study design such as dental hygiene practices, fluoride usage and dietary factors, including as the intake of sugar-based foods or beverages and non-cariogenic foods such as milk and dairy products. A systematic review assessing the relationship between obesity and dental caries concluded that there is limited evidence on this public health issue and well-designed longitudinal studies are required to demonstrate the relationship [23]. In addition, there have been no longitudinal studies on an Australian child population assessing the relationship between obesity, breastfeeding and ECC.

Our proposed longitudinal cohort study will assess whether there is an association of obesity and early childhood feeding practices with dental caries in preschool children after controlling for confounding variables such as fluoride use, sugar consumption and the intake of non-cariogenic food.

## Methods/Design

**Figure 1:**
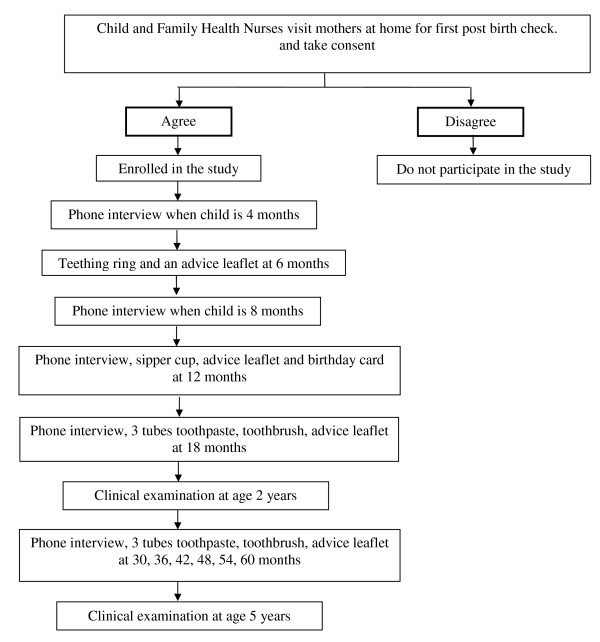
**Flow diagram of the recruitment strategy for the cohort study and data collection procedures**.

This cohort study is based in South Western Sydney to gather information on early childhood feeding practices and will assess their relationship to Early Childhood Caries (see Figure 1).

The aims of this longitudinal study are to:

1. Determine the association of breastfeeding and oral health of preschool children living in disadvantaged areas of NSW.

2. Identify the strength of association between ECC and putative risk and protective factors including socio-demographic influences, environmental (fluoride exposure) and behavioural factors (such as feeding and oral hygiene practices).

3. Identify the association between obesity and dental caries.

4. Quantify the longitudinal relationship between these factors on the progression of ECC from age 2 to 5 years

The specific hypotheses to be tested in the longitudinal study are:

1. Breastfeeding is associated with a lower number of white spot lesions and reduced decayed, missing and filled teeth (dmft) at 2 and 5 years of age.

2. Lower exposure to fluoride (water or toothpaste) is associated with higher rates of white spot lesions and decayed, missing and filled teeth at 2 and 5 years of age.

3. Poor health behaviours (such as frequent consumption of sweetened beverages, infrequent oral hygiene practices, night time bottle feeding) will be associated with higher rates of white spot lesions, decayed, missing and filled teeth at 2 and 5 years of age.

4. Children who are overweight or obese will have higher rates of white spot lesions and decayed, missing and filled teeth at 2 and 5 years of age.

Ethical approval to conduct this study has been granted by the Research Ethics Review Committees of the Sydney South West Area Health Service - RPAH Zone (ID number X08-0115) and the University of Sydney.

### Sampling design and recruitment

Sydney South West Area Health Service (SSWAHS) covers a number of locations where the population is socially disadvantaged. The children will be recruited from SSWAHS, soon after birth, when the Child and Family Health Nurses undertake the first home visit as this is the primary point of health professional contact for newborn children and their careers/parents [29]. The nurse unit managers (personal communication 2009) report that 95 percent of the mothers are seen for a post birth visit. The nurses in SSWAHS are concerned that dental disease is a serious problem which impinges on their working lives and wish to collaborate with this study. Pilot study data showed that gaining the informed consent at this time is an efficient way of recruiting mothers with young children.

### Eligibility

All children will be eligible to participate however, at the statistical analysis stage participants will be excluded from the study if there is presence of physical or intellectual disability in a child which is likely to influence dietary behaviours, hygiene practices and physical activity. Interpreter services will be arranged for non-English speaking parents and language appropriate written materials will be provided

### Sample Size

We calculated the minimal sample size required to achieve the aim 1, the most sensitive aim. In the Sydney South West Area approximately 40 percent of children aged 5 years have experienced caries [8]. It is anticipated that different feeding patterns would be associated with 15 percent change in caries experience at age 5 years. Sample size was calculated to a power of 90 percent and alpha of 0.05 (two-tail). A sample size of 560 children is required in this longitudinal study for dental examinations at 2 and 5 years. To accommodate for a 50 percent dropout over 5 years, the sample size has been increased to 900, and this mirrors other cohort studies [30,31]. This sample size would be adequate to achieve other aims with statistical power over 90 percent.

### Quantitative Data collection

At age 4, 8, 12, 18, 24, 30, 36, 42, 48, 54 and 60 months, a research assistant will call the families to update their socio-demographics details, complete an interview on feeding habits and oral hygiene practices. Selected questions from the first and second Perth Infant Feeding Studies [30,31] on childhood feeding patterns will be used to identify infant feeding practices. These include questions on duration and exclusivity of breastfeeding, duration of formula feeding and age of introduction of complementary foods (i.e. solids and non-milk beverages). In addition, questions related to nocturnal milk feeding have been added and a short food frequency questionnaire has been developed and tested to assess the intake of non-milk extrinsic sugars (cariogenic foods) [32] and dairy products (non-cariogenic foods) [33]. In addition, a validated early childhood oral health impact scale will be used at age 2 and 5 years to evaluate oral health related quality of life [34] (see Table 1). Care will be taken not to motivate a mother towards a specific feeding method or provide her with information on oral hygiene through telephone interviews.

**Table 1:** The Early Childhood Oral Health Impact Scale (ECOHIS)^34^

Problems with the teeth, mouth or jaws and their treatment can affect the well-being and everyday lives of children and their families. For each of the following questions please circle the number next to the response that best describes your child's experiences or your own. Consider the child's entire life from birth until now when answering each question. If a question does not apply, check 'Never'
Response options: 1. Never, 2. Hardly ever, 3. Occasionally, 4. Often, 5. Very often and 6. Don't know.
***Child Impacts***
How often has your child had pain in the teeth, mouth or jaws?
How often has your child ....because of dental problems or dental treatments?
had difficulty drinking hot or cold beverages
had difficulty eating some foods
had difficulty pronouncing any words
missed preschool, daycare or school
had trouble sleeping
been irritable or frustrated
avoided smiling or laughing
avoided talking
***Family Impacts***
How often have you or another family member member......because of your child's dental problems or treatments?
been upset
felt guilty
taken time off from work
How often has your child had dental problems or dental treatments that had a finacial impact on your family?

### Sample Retention Programme

At age six months a teething ring will be posted to all families together with language appropriate written advice on coping with the eruption of the baby teeth. Information on not adding sugar to milk formula will be provided and avoiding continuous use of a bottle as a comforter will be stressed. At twelve months a second leaflet prepared by NSW Health, advocating the use of sipper cups, will be sent to all the families, together with a birthday card. At 18 months, and thereafter at six monthly intervals until 5 years of age, all participants will receive child strength fluoride toothpaste, a toothbrush and dental advice leaflets. An annual birthday card for the children will be used to maintain interest in the study and minimise attrition. The products and leaflets were recommended by Nurse Unit Managers as one way of maintaining interest in the project. In addition, providing postal information on oral health will make the information available to the entire sample than to provide to particular at-risk families. If further funding is available other incentives may be used to reduce the dropout rate.

### Qualitative Data collection

Ten percent of randomly selected families, according to their first language, will be invited to complete an in depth semi-structured interview at their home when the child is one year old. All the interviews will be conducted by an experienced language appropriate researcher in qualitative research and will be tape-recorded. The focus of the interviews will be on issues around the child's feeding experiences.

### Outcomes measures

The proposed primary and secondary outcome measures at age 2 years and 5 years are shown in Table 2.

**Table 2:** Outcome measures for the cohort study

Phase One : Initial phase (0 to 2 years)		Phase Two: Follow up phase (3-5 years)
***Primary Measures***		
Dental caries prevalence at age 2 years		Dental caries prevalence at age 5 years
Oral health related quality of life		Dental caries incidence from age 2 to 5 years
Oral debris		Oral health related quality of life
		Oral debris
***Secondary Measures***		
Mothers knowledge/attitude on breastfeeding		Parental knowledge/attitude on preschool child oral health
Mothers experiences on breastfeeding		Parental opinions on the sample retention programme
Parental opinions on the leaflets		

### Clinical Examination

All children will be offered a free dental check up at 2 and 5 years. The primary outcome measure is the extent of dental caries and presence of oral debris on the primary teeth when the child is 2 and 5 years old. Teeth will be examined in order by quadrant (maxillary right, maxillary left, mandibular left, mandibular right) and surface (distal, occlusal, mesial, buccal and lingual). Criteria for caries examination will include cavitated (d2-d3) lesions, which require a demonstrable loss of enamel structure on visual examination or softness at the base of lesion on probing with a ball ended explorer; and non-cavitated (d1) lesions, characterized by an area of distinct chalky white enamel on a smooth tooth surface or adjacent to a pit or a fissure with no loss of enamel [35,36] (see Table 3). White spot lesions show early evidence of dental caries. Oral debris will be assessed by visual examination and if needed confirmed by wiping with a gauze (see Table 4). The examinations will be undertaken by trained and calibrated dental therapists. Repeat examinations will record intra examiner reproducibility and a gold standard examiner will be used to monitor inter examiner variation.

**Table 3:** Classification and codes used for dental caries examinations^36^

		**Non-cavitated (d**_**1**_**) Lesions**		**Cavitated (d**_**2-3**_**) Lesions**
***Smooth surfaces***				
Appearance/Colour		Chalky white		Chalky white with darker center
Surface		Intact		Cavitated - definite loss of tooth structure
Tactile		Normal (tactile exam usually not necessary)		Soft
Location		Usually adjacent to soft tissue margin		Usually adjacent to soft tissue margin
***Pits and Fissures***				
Appearance/Colour		May be lightly stained, or have chalky white		Oftens stained light to dark brown and
		area adjacent to pit or fissure		often with chalky white area adjacent
Surface		Intact		Cavitated - definite loss of tooth structure
Tactile		Normal		Soft
Undermining		Not present		Often evident

**Table 4:** Classification and codes used for oral debris examination

Assessment	Code	Explanation
Debris index	0	Absence of debris/plaque
	1	Plaque/debris visible

Dental Assistants will be trained in anthropometric techniques to collect height, weight and waist measurements of the children at ages 2 and 5 years.

### Quantitative Data Analysis

Analysis will be conducted at the individual level using SAS version 9. Variations in dental caries experience indices (dmfs, dmft) will be analysed in relation to the following independent variables: duration, frequency and exclusivity of breastfeeding and nocturnal milk feeding patterns (frequency and type of milk i.e. breast milk or formula), and Body Mass Index (BMI). BMI will be calculated as weight (kg)/height (meters) squared and children at age 2 and 5 years will be classified as not overweight or obese, overweight or obese, based on the International Obesity Taskforce recommended age-standardised BMI cut points [37]. These will be adjusted for other dietary factors such as cariogenic and non-cariogenic foods, fluoride use and socio-demographic factors. The prevalence rate ratios at age 2 and 5 years and their 95% confidence intervals (CI) will be calculated and will be used to evaluate their corresponding incidence rates. The effects of the independent variables on caries prevalence and incidence will be quantified through logistic regression analyses.

Potential risk factors associated with the development of caries from age 2 to 5 years will be assessed by incidence density ratio and odds ratios through logistic regression analyses [38-40].

Associations between the measures of oral health status (dmft, dmfs) at ages 2 and 5 years and the yearly responses to the early childhood oral health impact scale will be explored by correlation analysis.

### Qualitative Data Analysis

The interviews will be transcribed and entered in and analyzed with the assistance of NVivo 9.0 qualitative analysis software. Content analysis as recommended for qualitative descriptive studies will be used [41]. Two research team members will review each transcript and assign codes to meaningful units of data (such as sentences, phrases), and then compare and reach a consensus and these will be used to illustrate themes.

## Discussion

Prevention and management of Early Childhood Caries (ECC) should begin early in life, as this public health problem is evident in children as young as 2 years of age. To date, there are no longitudinal studies in Australia on the association of breastfeeding, obesity and the oral health of preschool children. This study will provide robust evidence on infant feeding practices (exclusive breastfeeding, breastfeeding duration and nocturnal breastfeeding) and BMI and its relationship to oral health of young children. It will also provide useful information on potential predictors for dental caries in young children.

Repeated interaction with the caregivers on the telephone and the provision of advice and products may influence oral health behaviours. However, the success rates for many health promotion programs are low, and those that are successful usually have sustained interventions with considerable follow up. We are therefore assuming our intervention to maintain interest in the cohort study may have some effect, but will not have a major impact on behaviour influence. In addition, we will compare caries rates of our study population with that of state-wide data.

The strengths of this study include an innovative socio-ecological approach; utilization of a postal delivery of free products to minimize attrition; collection of longitudinal data to monitor feeding patterns and prepare explanatory models to link them with dental caries; and the focus on disadvantaged families for whom prevention programmes are a public health priority.

## Competing interests

The authors declare that they have no competing interests.

## Authors' contributions

AA, ASB and JAS conceived the project. All authors participated in its design and methodological development. AA drafted the initial manuscript. All authors commented on the drafts and approved the final manuscript. ASB and SB contributed to the procurement of funding. SB contributed to gaining access to SSW Oral Health and AHS resources.

## Pre-publication history

The pre-publication history for this paper can be accessed here:

http://www.biomedcentral.com/1471-2458/11/28/prepub
